# *Legionella* in the City: Unveiling *Legionella pneumophila* in Hillbrow’s High-Rise Water Systems

**DOI:** 10.3390/microorganisms13092152

**Published:** 2025-09-15

**Authors:** Keletso Emily Buthane, Zaakirah Delair, Tobias George Barnard, Atheesha Singh

**Affiliations:** Water and Health Research Centre, University of Johannesburg, Doornfontein, Johannesburg 2028, South Africa; keletsobuthane@gmail.com (K.E.B.); zdelair@uj.ac.za (Z.D.); tgbarnard@uj.ac.za (T.G.B.)

**Keywords:** *Legionella pneumophila*, free-living amoeba, Legiolert

## Abstract

Urban settings in developing countries present unique challenges such as high population density, inadequate water infrastructure and water supply, all factors that contribute to the growing threat of premise plumbing pathogens such as *Legionella*. Water droplets from showers and faucets aerosolise *Legionella*, which, when inhaled, invade the human respiratory tract to manifest as Legionnaires’ disease. Densely populated, high-rise buildings present an ideal case study for investigating the presence of *Legionella.* The aim of this study was to investigate the occurrence of *Legionella pneumophila* (*L. pneumophila*) in water systems of 15 high-rise buildings in Hillbrow, Johannesburg, South Africa. A total of 67 hot- and cold-water samples and 121 swab samples were collected and analysed for the presence of *Legionella pneumophila.* Samples were analysed using the Legiolert assay, the South African National Standard (SANS) 11731:2017 method, and the amoeba enrichment method for detecting amoeba-associated *Legionella.* Molecular confirmation of the pathogen was conducted using conventional PCR and quantitative real-time PCR targeting the *mip gene. Legionella pneumophila* was found in 93% (14/15) of the buildings that were sampled and was more prevalent in cold-water samples (65%) compared to warm-water (35%) samples. All buildings were positive (100%) for the growth of free-living amoeba (FLA) from water and swab samples. Of these samples, three were confirmed positive for *L. pneumophila* by PCR and the sequencing alignment results confirmed the identity and relatedness of the isolates to *L. pneumophila*.

## 1. Introduction

*Legionella pneumophila* (*L. pneumophila*) is an opportunistic, Gram-negative pathogen that was first discovered during a pneumonia outbreak in 1976. It has since been shown to be responsible for several other disease outbreaks marking its significance in public health [1,2]. Infection by this bacterium follows inhalation of aerosolised water droplets that are contaminated with the bacterium [3], which then invade the human lungs and manifest in a pneumonic disease known as Legionnaires’ disease (LD) or a milder, non-pneumonic form called Pontiac fever [4,5]. Advanced age (age ≥ 50), smoking, diabetes, cancer, immune suppression, chronic lung disease, iron overload and alcohol misuse are factors that predispose a patient to infection [1,6,7]. Other *Legionella* species such as *L. longbeacheae*, *L. bozemanii* and *L. dumoffii* have also been implicated in manifesting LD [8,9].

This pathogen exists in freshwater ecosystems as a free-living microbe, within a parasitic host or as part of a biofilm [10,11]. In an environmental setting, it is more often incorporated into an already existing multi-species biofilm that increases its chances of survival, spread, resistance to disinfection and even virulence [12]. The replicating cycle begins when the bacterium is engulfed by phagocytic alveolar macrophages or free-living amoebae (FLA), where it employs mechanisms to evade phagocytosis before the release into the environment of progeny with a relatively higher stress resistance and virulence. The cycle begins anew when these microbes encounter another host, or they enter bulk water supply systems and become aerosolised [13]. The ability of the bacterium to survive in a wide range of environmental conditions, coupled with its presence in engineered water systems, underscores the importance of regular water monitoring and control measures to prevent outbreaks. This is particularly crucial in healthcare settings and places with large, complex water systems, where vulnerable populations may be exposed to the pathogen [14].

Common detection methods for the enumeration and detection of *Legionella* include culture-based methods such as Standard Methods 9260: *Legionella* method [15,16] or the ISO 11731:2017 [17], which is the gold standard in plate culturing [18]. Samples are cultured on Glycine–Vancomycin–Polymyxin–Cycloheximide (GVPC) or Buffered Charcoal Yeast Extract (BCYE) agar with L-cysteine and iron salts, which are essential for the growth of the bacterium. The standard method requires an added step of latex agglutination and cysteine dependency for further confirmation [19,20]. An alternative detection method is the Legiolert method, an enzyme–substrate assay which targets *L. pneumophila* while restricting growth of its commensals. The Legiolert method received validation NF for the AFNOR (Association Française de Normalisation) certificate (AFNOR, No.: IDX 33/06-06/19) with comparative results to the ISO 11732-2017 [21]. Other detection methods, such as direct fluorescent antibody staining and enzyme-linked immunosorbent assay, are employed for the detection of *L. pneumophila* serogroups in clinical samples [16]. The addition of molecular methods such as PCR provides higher sensitivity and specificity when detecting the bacterium [22].

Upon entering a building’s water system, favourable conditions for *Legionella* growth include water temperature, water stagnation, and the presence of biofilms and FLA [23]. The abundance of FLA in water systems provides a nutrient-rich refuge that allows them to proliferate and grow freely and has led to the ineffectiveness of antimicrobial control agents towards *Legionella* in fresh drinking water [24,25]. Understanding the mechanism of infection and disease progression is crucial for developing effective prevention and control strategies.

The densely populated Hillbrow metropolitan area of Johannesburg, South Africa, consists of 24,857 households, with about 10,000 flats, motels and free-standing houses. Many of these buildings face challenges such as maintenance issues, overcrowding and inadequate infrastructure exacerbated by a high rate of urbanisation, which greatly affects the spread and response to diseases, and subsequently the health of residents [26]. In addition, socio-economic factors such as poverty and unemployment and the increasing phenomenon of occupation of abandoned buildings and illegal access to public services, create a strain on resources and infrastructure [27]. The state of such buildings makes it a possible premise plumbing environment with predisposing factors that may promote opportunistic premise plumbing pathogen (OPPP) colonisation and growth, and consequently the risk of LD for the residents. Outbreaks in hospitals and other public areas are the only investigated incidences of *Legionella* in South Africa, resulting in a relative neglect of this infection in single households [28].

Thus, although LD is a notifiable disease as stipulated by the Occupational Health and Safety Act, No. 85 of 1993, it is rarely reported in South Africa [28]. *Legionella*’s ability to form resilient biofilms, associate with amoeba and resist disinfectants necessitates regular, routine water testing as well as comprehensive surveillance and control measures to safeguard public health, particularly for vulnerable populations. Since this pathogen thrives in complex water systems, posing significant public health risks, especially in densely populated areas, this study highlights the need for *Legionella* monitoring in building water systems as well as a consolidation of detection methods for the accurate representation of the pathogen in a water system.

## 2. Materials and Methods

This was a cross-sectional descriptive study that investigated the direct occurrence of *L. pneumophila* as well as *Legionella* in amoeba from water and swab samples obtained from buildings in Hillbrow, Johannesburg.

### 2.1. Study Setting

**Figure 1:**
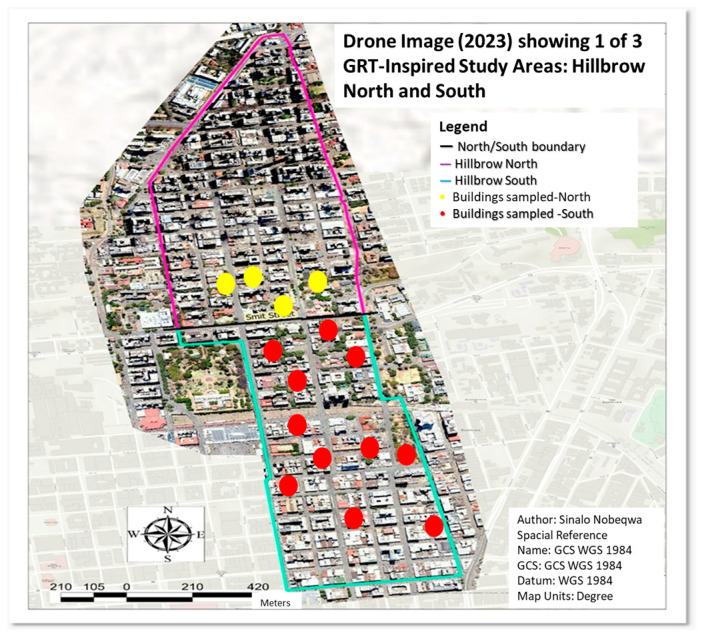
Map of Johannesburg inner city showing the Hillbrow high-rise buildings sampled in this study represented as yellow dots (buildings in the north Hillbrow region) and red dots (south Hillbrow region) in the location. The map was adapted from the Gauteng Research Triangle—South African Population Research Infrastructure Network (GRT-SAPRIN) of South Africa [29].

The selected study site was in the south of Hillbrow, situated near the inner city of Johannesburg, Gauteng (Figure 1). These buildings have old infrastructure and accommodate low-income and working-class individuals and families. Access to the study site was approved through the Gauteng Research Triangle Initiative for the Study of Population, Infrastructure and Regional Economic Development (GRT-INSPIRED). Water samples were only collected from households that agreed to participate in the study and provided safe access for water collection. Ethical clearance was granted by the University of Johannesburg Research Ethics Committee, REC: 1656-2022.

### 2.2. Sample Size

For the study, convenience sampling was used, and fifteen (*n* = 15) high-rise buildings from the Hillbrow region were selected. A total of 67 apartments were sampled for water and biofilms during November 2022, February, March, April and May 2023 (December and January are transitional periods for residents in Hillbrow). To retain anonymity and confidentiality, the buildings were coded from HB1 to HB15.

### 2.3. Building Walkthrough Assessment

Upon approval and consent, building walkthrough assessments were conducted on-site and available residents were contacted to participate in the study. The building walkthrough assessments were conducted using a checklist (Supplementary Material Table S1) to determine the general building information (age of building, number of apartment units, and occupancy of building); water management and distribution in the building (water quality, distribution and type of plumbing material used); and possible aerosolisation points, such as availability of toilet seats, air ventilation and stagnation points. The quality of water was primarily assessed through taste, smell and colour in accordance with Clause 7 of the South African National Standard (SANS) Number 241 [30] that encodes for aesthetic water quality for visual, aromatic and palatable water quality standards.

### 2.4. Sample Collection

Three litres of each water sample (sufficient for all three methods used to detect the pathogen) were collected post-flush from the top, middle and bottom floor apartments of each study building. As indicated in Table 1, water samples were collected from bathroom faucets, while swab samples were collected from bathroom faucets and showerheads (where available), toilet bowls and storage tanks. Hot water (*n* = 23) was prioritised, but where the apartment had no available hot water, cold water (*n* = 44) was collected instead, to assess if the circumstances affect the occurrence of *L. pneumophila*. Sampling bottles contained 1 mL of 0.1 N sodium thiosulfate solution to neutralise residual chlorine. Biofilm samples (*n* = 121) were collected using sterile swabs containing 6 mL transport medium (salts, buffers and a gelling agent) (Cary-Blair Collection and Transport System, Copan Diagnostics, Inc., Murrieta, CA, USA). Separate swabs were then inserted into tap (faucet) outlets, showerheads, toilet bowls and storage tanks before being returned to the swab transport medium and kept at 4 °C until analysis. Water processing for Legiolert (IDEXX) and Colilert (IDEXX) Quanti-Trays was performed immediately upon arrival at the laboratory, whilst the direct culture (ISO 11731) and amoeba cultivation were performed within 24–48 h after arrival (within the maximum acceptable storage time recommended by ISO 19458 [31]). All samples were kept at 4 °C until analysis.

### 2.5. Physico-Chemical Parameters

Physico-chemical parameters such as temperature, pH, conductivity and total dissolved solutes (TDS) were tested onsite using a portable Combo Tester^®^ (Hanna instruments, Woonsocket, RI, USA, HI98129). From each collected sample, an aliquot was poured into a sterile 125 mL red cap sample container (Thermo Scientific, Johannesburg Gauteng, South Africa) to test for these parameters. The probe was rinsed with sterile distilled water before and after contact with each sample. Residual and total chlorine were tested onsite using a chlorine photometer (YSI 900 Single Parameter Colorimeter, YSI, Yellow Springs, OH, USA). Turbidity was measured using the Oakton DW-35635-00 T-100 turbidity meter (EUTECH, Tuas, Singapore) at the laboratory.

### 2.6. Detection of Escherichia coli and Total Coliforms

The IDEXX Colilert Quanti-Tray/2000 assay (IDEXX, Midrand, South Africa) was used to detect the presence of faecal coliform *Escherichia coli* (*E. coli*) and total coliforms. Each 100 mL sample was processed as per the manufacturer’s instructions, and each sample set included controls such as *Klebsiella pneumoniae* (ATCC No. 31488) as a positive coliform indicator, *E. coli* (ATCC No. 25922) as a faecal coliform positive indicator, *Pseudomonas aeruginosa* (ATCC No. 27853) as a negative control and sterile distilled water as a product negative control. As per the manufacturer’s instructions, wells that fluoresced under a UV light were deemed positive for *E. coli*, while a yellow colour in the wells was positive for coliforms. The Most Probable Number (MPN) of each tray was calculated using the MPN table provided (IDEXX, Midrand, South Africa).

### 2.7. Detection of Legionella pneumophila by the Legiolert Quanti-Tray Assay

Each 100 mL water sample was used to analyse the presence of *L. pneumophila* using the Legiolert Quanti-Tray assay (IDEXX, Midrand, South Africa) according to the manufacturer’s instructions for drinking water [31]. The hardness of the water sample was determined with a hardness test strip (Aquadur^®^ hardness test strip, IDEXX, Midrand, South Africa) supplied with the Legiolert kit, and an appropriate measure of the Legiolert supplement (IDEXX, Midrand, South Africa) was added according to low or high hardness of the water as per the manufacturer’s instructions. All analyses included a positive control (*Legionella* ATCC 33152) and *Enterococcus faecium* (ATCC 29212) as a negative control and autoclaved distilled water as a product control. After incubation, positive results were determined by turbidity and/or a brown colour change in the wells. The number of positive wells was counted, and the MPN was determined using the *Legionella* MPN table supplied by the manufacturer. From each Legiolert positive tray, a total of 2 mL of broth was collected from the positive wells for DNA extraction and PCR.

### 2.8. Detection of Legionella spp. by the South African National Standard—SANS 11731:2017 Method

The South African National Standard—SANS 11731:2017 for detection and enumeration of *Legionella* [32] was used to detect *Legionella* from the water and swab samples. A total of 100 mL of each water sample was filtered through a sterile 0.45 µm nitrocellulose membrane. Each membrane was acid-treated (acid prepared according to the SANS 11731:2017 protocol) and washed with Page’s Amoeba Saline (PAS) (Oxoid, Hampstead, UK) (as per the SANS 11731:2017 method) before being placed on a plate of BCYE agar (with supplement). The agar plates were incubated at 37 °C for up to 10 days. Each swab sample was transferred into individual sterile tubes containing 10 mL PAS (Oxoid, UK) and vortexed (Lasec, Cape Town, South Africa) for 30 s at maximum speed. Then, 1 mL of each suspension was inoculated onto a BCYE (with supplement) agar plate. Grey/white, glass-like presumptive colonies (as described in SANS 11731:2017) were then sub-cultured onto fresh BCYE with supplement plates and nutrient agar plates and incubated at 37 °C for 24 h to check for cysteine dependency. Colonies that grew on BCYE agar but not on nutrient agar were considered cysteine-dependent and thus positive for *Legionella.* These colonies were inoculated into Lennox broth (made up of 10 g/L Tryptone, 5 g/L Yeast extract, 5 g/L sodium chloride) with the BCYE supplement added to promote growth of *Legionella* and incubated at 37 °C for 48 h. The broth was used for DNA extraction and PCR.

### 2.9. Detection of Amoeba-Associated Legionella

To investigate the presence of amoeba-associated *Legionella,* amoebae were detected in water samples [33]. Each water sample (500 mL) was filtered through a sterile 0.45 µm nitrocellulose membrane (Millipore, Johannesburg, South Africa) and the membrane was placed on a Non-Nutrient Agar plate (NNA) (Oxoid, UK), overlaid with a 100 µL suspension of heat-killed *E. coli* (*E. coli* ATCC No: 25922). Each suspension was placed for 20 min in a 100 °C water bath. For amoeba detection from swab samples, 1 mL of the swab suspension (above) was added onto NNA plates prepared with heat-killed *E. coli* and incubated at 32 °C for 2 weeks. A light microscope (Olympus, Tokyo, Japan) was used to examine the plates every three days for the presence of FLA trophozoites and/or cysts. Samples showing the presence of amoebae (at the trophozoite and/or cyst state) were sub-cultured and incubated at 32 °C for 72 h. Once purified, the cells were suspended in PAS and washed by centrifuging (Vacutec, Bolton, UK) at least three times at 7500 rpm for 15 min. Giemsa staining was used for visualisation under the microscope. Briefly, a smear of the suspension was prepared by heat fixing onto a slide and flushed with 20% Giemsa stain solution (20 mL of Giemsa stock solution mixed with 80 mL of sterile distilled water, pH 7). After 10 min, each slide was washed with water and left to dry. A drop of immersion oil was added onto the dry slide and visualised under a 100× lens on a light microscope (Olympus, Tokyo, Japan). The washed presumptive amoebae were passed through a sterile syringe needle (21 G, Sterican, Léry, QC, Canada) to lyse the amoebae and to release intracellular bacteria. The lysate was then cultured on BCYE media with supplements and incubated at 32 °C for 24 h. Presumptive *Legionella* colonies were inoculated onto Lennox broth (as described above) and incubated at 32 °C for 24 h. From the broth culture, 2 mL was used for DNA extraction and PCR targeting *Legionella* spp. and *L. pneumophila* with primers described in Table 1. Samples that were positive for the presence of amoeba were selected for DNA extraction, and common FLA PCR was performed using FLA primers outlined in Table 2.

### 2.10. DNA Extraction

Broth from the Legiolert (2 mL), Lennox broth (2 mL) and amoeba lysate were used for DNA extraction using the Guanidium Thiocyanate DNA extraction method, as reported by Omar and Barnard [34]. DNA was eluted in 100 µL PCR water, of which 2 µL was used as a template in the PCR reaction.

**Table 2:** Primer sequences and cycling conditions used to detect *Legionella* species, *Legionella pneumophila* and free-living amoeba.

Primer Name	Sequence 5′–3′	Size (bp)	Cycling Conditions	References
Leg 225 (F)	AAGATTAGCCTGCGTCCGAT	658	95 °C for 15 min, 95 °C for 1 min, 55 °C for 90 s, 72 °C for 1 min, (30 cycles) 72 °C for 10 min	[35]
Leg 858 (R)	GTCAACTTATCGCGTTTGCT			
*L. pneu* (F)	CCGATGCCACATCATTAGC	150	95 °C for 15 min, 95 °C for 30 s, 55 °C for 30 s, 72 °C for 5 min, (34 cycles)	[36]
*L. pneu* (R)	CCAATTGAGCGCCACTCATAG			
PFLA (F)	CAGGTTAAGGTCTCGTTCGTTAAC	750–1000	95 for 15 min, 94 for 1 min, 62 for 90 s, 72 for 1 min, 72 for 10 min (34 cycles)	[37]
PFLA (R)	CAGGTTAAGGTCTCGTTCGTTAAC			
*L. pneu*	CCGATGCCACATCATTAGC	150	95 °C for 15 min, 95 °C for 30 20 s, 60 °C for 60 s (43 cycles)	[38]
	CCAATTGAGCGCCACTCATAG			
Probe	5′6-carboxyfluorescein (FAM)-TGCCTTTAGCCATTGCTTCCG-BHQ			

### 2.11. Legionella PCR

Samples were tested for the presence of *Legionella* spp. and *L. pneumophila* using the primer sets described in Table 2. Each *Legionella* PCR amplification reaction was performed in 20 µL reaction mixture containing 2 µL of the 10× PCR Buffer, 0.1 µL HotStar *Taq* DNA polymerase, 1 µL 5× Q-solution, 0.6 µL (10 mM each) dNTP mix, 1 µL (10 µM) each of forward and reverse primer, 2 µL of template DNA, 2 µL (25 mM) MgCl_2_ and 10.3 µL PCR-grade water. The PCR was performed in a T100^TM^ thermal Mycycler (BIORAD, Roodepoort, South Africa) with specific conditions as described in Table 2. All amplicons were separated on a 2% (*w*/*v*) agarose gel made with TAE buffer and viewed under UV light using the Omega Fluor acquisition software version 2.0.1013.0 (Vacutec, UK). The relevant sizes of the DNA fragments were determined by comparing their electrophoretic mobility to that of a standard 1 Kbp marker (Fermentas^®^, Charlestown, MA, USA) that was run with the samples on each gel.

### 2.12. Real-Time Quantitative PCR

The samples that were positive by conventional PCR were then confirmed by real-time PCR using the primer-probe sequence and cycling conditions as described in Table 2. Amplification by real-time PCR was performed in each of 20 µL reaction mixtures containing 2 µL of the 10 × PCR Buffer (QIAGEN^®^ Hotstart *Taq* DNA polymerase and 15 mM MgCl_2_), 0.6 µL (20 mM each) dNTP mix, 2 µL (10 µM *LpneuF, LpneuR, LpneuP* probe mix), 2 µL of template DNA, 0.1 µL HotStart *Taq* DNA polymerase, 2 µL MgCl_2_ and 11.3 µL PCR-grade water. The extracted DNA was amplified in a Rotor-Gene Q (QIAGEN, Venlo, The Netherlands).

A standard curve was generated using 10-fold serial dilutions of known *L. pneumophila* DNA concentrations in triplicate. The standard curve showed a strong linear relationship (R^2^ = 0.99) with a lower level of detection (LLD) of 11.89 copies/reaction (Ct = 24.04) and an upper level of detection (ULD) of 19.52 copies/reaction (Ct = 16.26). Samples with Ct values higher than the LLD (i.e., Ct > 24.04) and lower than the ULD (i.e., Ct < 16.26) were excluded from analysis.

### 2.13. Sequencing

Isolates that tested positive by PCR for the amoeba-associated *Legionella* were sent for sequencing to Inqaba Biotech (SA). These samples were analysed using *L. pneumophila* primers targeting the *mip* gene, which is crucial for *L. pneumophila* virulence. The resulting sequences were entered into the NCBI database for BLAST (version 2.17.0) analysis to identify similarity or high homology to known *Legionella* sequences.

### 2.14. Data Analysis

A Wilson 95% confidence interval test, Spearman rank-based test and Pearson (Linear) test were used to assess the correlation between *Legionella* positivity and the water source. Bivariate analysis was used to assess the relationship between the temperature of the water and *Legionella* positivity. Significance was set at *p* < 0.05. Statistical analysis and preparation of graphs used GraphPad Prism v10.

## 3. Results

### 3.1. Building Walkthrough Assessment

A building walkthrough assessment was used to classify the building type according to SANS 10400-A:2022 [39]. From the 15 buildings sampled, 87% (13/15) were domestic residences and categorised as class H3 (domestic residence), whilst 13% (2/15) fell under class H5 (Hospitality). Within class H5, one building was a communal student residence, and the other was a residential building that also doubled as a hotel.

**Figure 2:**
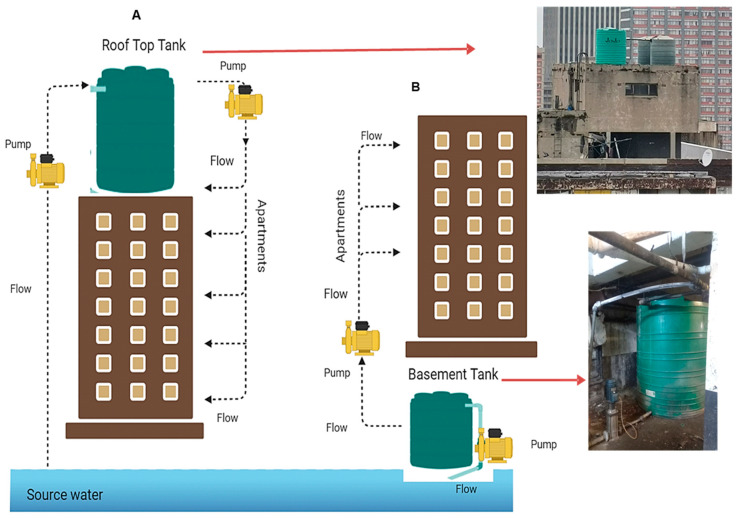
Water distribution systems in the Hillbrow high-rise buildings. (**A**) Flow of water from the source of water to a rooftop tank and back down to the apartments, (**B**) flow of source water from the basement tank up to the apartments.

While the ages of all the buildings were unknown, renovations to some (40%; *n* = 6) of the plumbing systems were relatively recently performed (between 2019 and 2020), while records of such maintenance were not available for the remaining buildings (60%; *n* = 9). The buildings ranged from 4 to 14 floors, with 10 of the buildings having occupancy ranging from 50% to 65%, four buildings having 90 to 97% occupancy, and one building being 100% occupied (H8), as indicated in Supplementary Material Table S1. All the buildings employed a storage tank in their drinking water distribution system. The location of the storage tanks for 60% (9/15) of the buildings was on the roof, where water was pumped from the source up to the roof before flowing down to the residences. Alternatively, the storage tank was located at the bottom level of the building from which water was pumped up to the residences, as was the case for 40% (*n* = 6) of the buildings, as shown in Figure 2. The material used to manufacture the storage tanks was plastic in 93% (*n* = 14) of the buildings, whilst the remaining buildings had a metal storage tank. The plumbing material was unspecified in 53% (*n* = 8) of the buildings, with 27% (*n* = 4) using iron, 7% (*n* = 1) using copper, whilst 13% (*n* = 2) used a combination of both copper and polyvinyl chloride (PVC).

**Figure 3:**
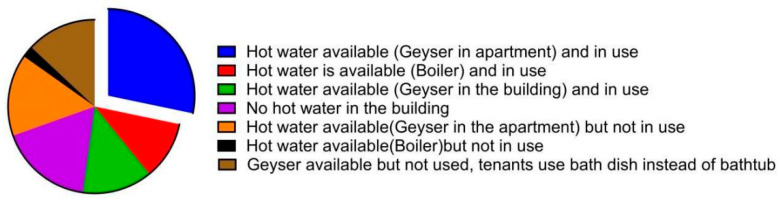
Hot water availability and use per sampled apartment. Note that responses regarding water use were made at the discretion of the tenants.

As indicated in Figure 3, the availability of hot water differed per building and was subject to the hot water distribution system that the buildings employed. Water was heated continuously throughout the day in 27% (*n* = 4) of the buildings. This was due to the availability of a central hot water heater (e.g., boiler). Although 60% (*n* = 9) of the buildings had geysers per apartment, water heating schedules differed and were at the discretion of the tenant. No water heating equipment was found in 13% (*n* = 2) of the buildings.

Occupants of all (*n* = 15) study buildings declared that the water had no unusual taste or smell, and the colour was clear. Of these buildings, 7% (*n* = 1) had an open storage tank, most (87%; *n* = 13) relied on a municipal water supply, and 13% (*n* = 2) had a man-made borehole as a drinking water source. Nearly a third of the buildings (33%; *n* = 5) had a means of onsite secondary water treatment, while 7% (*n* = 1) of the buildings only employed on-site water treatment when deemed necessary. Most (60%; *n* = 9) of the buildings did neither. A water quality manager was assigned to only 27% (*n* = 4) of the buildings. Monthly water quality monitoring was conducted in only 40% (*n* = 6) of the buildings, while the remaining buildings did not monitor water quality, as shown in Table 3. All buildings had toilet seat covers in all their apartments, hence no points of aerosolisation were observed.

### 3.2. Physico-Chemical Parameters

**Figure 4:**
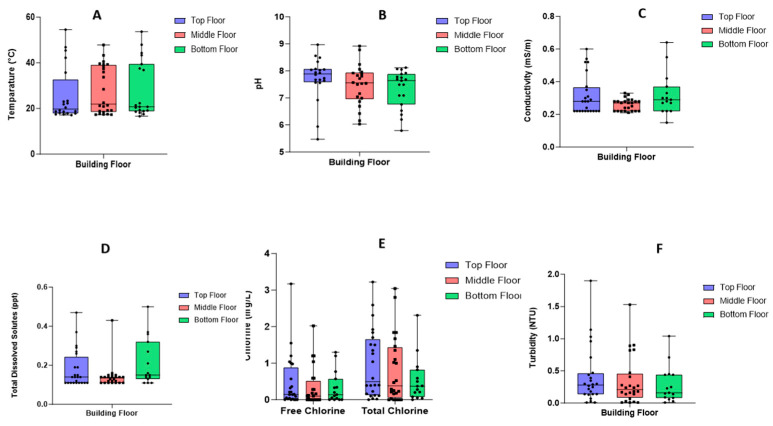
Box plot representing physico-chemical data comparisons per floor taken on the water samples: (**A**) represents the temperature (°C), (**B**) represents the pH, (**C**) shows the conductivity (µS/cm), (**D**) reflects the total dissolved solutes (ppt), (**E**) shows both the free and total chlorine (mg/L) and (**F**) show the turbidity of the samples.

A total of 23 hot water samples and 44 cold water samples were collected, of which 75% (*n* = 50) were from geysers, 18% (*n* = 12) were from a boiler, 6% (*n* = 4) were from storage tanks, while a single (*n* = 1) building obtained water from a borehole. The average pH of tested samples was 7.7 (min: 5.47; max: 9.0), and the samples collected from the buildings were within recommended pH levels, as shown in Figure 4B. Total chlorine levels for cold water averaged 1.43 mg/L (min: 0 mg/L; max: 3 mg/L), and for hot water, these levels averaged 0.76 mg/L (min: 0 mg/L; max: 2 mg/L). Figure 4E indicates a difference in total chlorine levels in water at various floors—total chlorine levels were higher at the bottom floor and levels reduced at higher floor levels. The free chlorine levels of cold water had a mean of 1 mg/L (min: 0 mg/L; max: 2 mg/L), and for hot water, the mean was 0.37 mg/L (min: 0 mg/L; max: 1 mg/L). Also, as indicated in Figure 4E, the free chlorine levels followed a similar pattern to that shown by the total chlorine levels. The temperature recorded for hot water between the buildings ranged from 30 °C to 55 °C, while the cold-water temperature ranged from 17 °C to 29 °C. The lowest water temperature was 17.1 °C, and the overall average temperature of water was 30 °C. Figure 4A indicates that most of the water was within acceptable ranges for potable water. The temperature versus chlorine readings for the buildings sampled are shown in Supplementary Material Table S2. The average turbidity was 0.34 NTU (min: 0.01 NTU, max: 1.9 NTU), and Figure 4F indicates that the turbidity of the samples was within acceptable ranges. The average conductivity was 0.26 µS/cm (min 0.15 µS/cm, max 0.33 µS/cm). Although all the samples were within acceptable ranges, Figure 4C indicates high ion levels at the bottom floor. The total dissolved solids (TDS) averaged 0.14 ppt (min: 0.11 ppt; max: 0.5 ppt), and Figure 4D indicates a high TDS at the bottom floor as well as levels at acceptable ranges.

### 3.3. Detection of E. coli and Total Coliforms

Results from the Quanti-Tray analysis indicated that 87% (*n* = 13) of the buildings had no coliform growth in their water systems, while the remaining buildings (*n* = 2) were positive for total coliforms, and of these, only one was positive for *E. coli* (3.43 MPN/100 mL).

### 3.4. Culture-Based Detection of Legionella pneumophila

**Figure 5:**
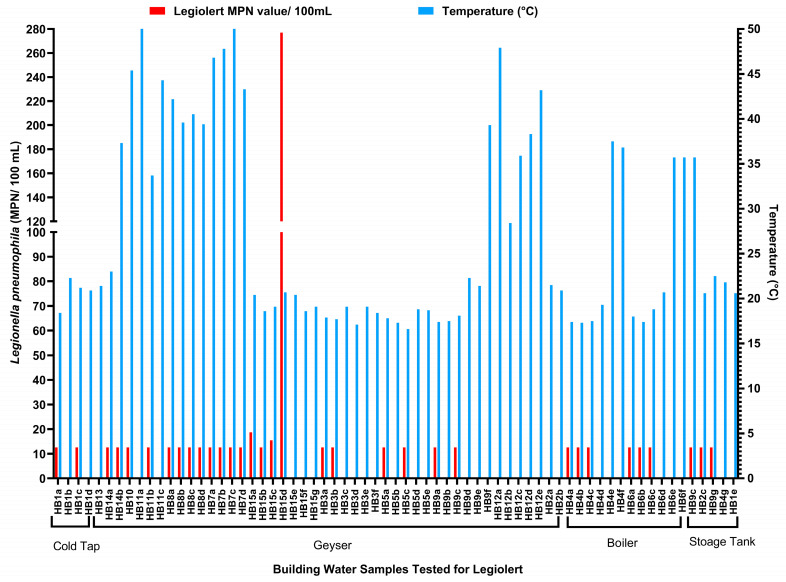
Bar graph illustrating the Legiolert MPN value (red) in relation to the water temperature (blue) and source (i.e., cold tap, geyser, boiler, and storage tank) collected from different apartments in the buildings (the lowercase letters indicate floors so that a: ground floor, b: lower middle floor, c: top floor /upper middle floor (higher buildings), d: top floor (shorter buildings, e: upper middle floor (higher buildings), f: Top floor/higher buildings, g: floor/higher buildings).

Analysis of results generated using the Legiolert method showed that *L. pneumophila* was detected in 14 out of the 15 buildings tested. As shown in Table 3, nearly half (46%; *n* = 31) of the water samples tested positive for *L. pneumophila* using the Legiolert assay, and of these, most (65%; *n* = 20) of the positives were cold water samples, and the remaining 35% (*n* = 11) were warm water samples. Of the 31 Legiolert-positive samples, 42% (13/31) tested positive for *Legionella*, and all (100%; *n* = 13) tested positive for the *L. pneumophila mip* gene. These data were confirmed with real-time PCR, where *Legionella* could only be detected in 1% (*n* = 2) of the water samples using the SANS 11731:2017 method. When the positive *L. pneumophila* samples were traced back to their water source, 74% (23/31) were from a geyser (cold and hot water), 19% (6/31) were from a boiler, and 7% (2/31) were from storage tank samples. These data are summarised in Figure 5.

### 3.5. Detection of Free-Living Amoeba and Amoeba-Associated Legionella

All the water (*n* = 67) and swab (*n* = 121) samples from the buildings (*n* = 15) were positive for amoeba growth. Some of the building (20%; *n* = 3) showed a higher amoeba presence in their water samples than in the swabs, whilst 27% (*n* = 4) of buildings had higher amoeba presence in swabs compared to the water samples and over half (53%; *n* = 8) of the buildings showed an equal presence of amoeba in both water and swabs. The results of the PCR confirmed the amoeba as FLA in 47% (*n* = 7) of the buildings.

**Figure 6:**
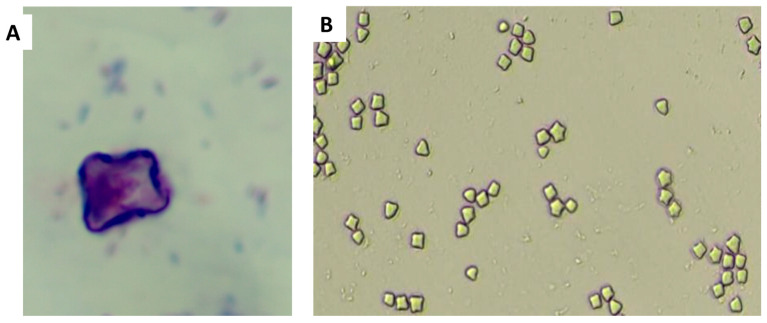
(**A**) Giemsa-stained image showing *Acanthamoeba* cyst (taken from a water sample) with endocytosed bacteria as well as bacteria outside the amoeba cyst (purple and blue colours, respectively), (**B**) *Acanthamoeba* cysts viewed under a microscope (40× magnification, Olympus Microscope, Tokyo, Japan).

Of the 67 collected water samples, 3% (*n* = 2) were positive for culturable *Legionella*, and 2% (*n* = 2) of the 121 swab samples cultured were positive for culturable *Legionella*. The PCR results showed that only three of the four (75%) of the samples were confirmed as *L. pneumophila*. These isolates were sent for sequencing. Figure 6 depicts different features of amoeba when viewed under a light microscope.

### 3.6. Sequencing

The DNA sequences were confirmed using BLAST (version 2.17.0) alignment against sequences in the NCBI database. Table 4 indicates that BLAST analysis showed a high specificity with 96–99% relatedness to the published *L. pneumophila* sequences.

### 3.7. L. pneumophila Positivity 

As indicated in Table 5, the precise Wilson 95% confidence interval (CI) analysis of water samples from the geyser (46.7%; 95% CI: 32.9–60.9) and boiler (50.0%; 95% CI: 25.4–74.6) highlights these as high-risk sources of *Legionella* colonisation. The limited number of samples for the other water sources presented a wider confidence interval and made it difficult to determine the true risk of these sources. This variability in low sample size highlights the need for caution when interpreting positivity rates. To reinforce the statistical positivity, odds ratio (OR) and 95% CI were estimated (Fisher’s exact test) relative to the geyser samples as a reference since the geysers had the largest number of samples collected and are generally recognised as a main source of *Legionella* contamination in building systems. Boilers (OR = 1.14, 95% CI: 0.34–3.80) and storage tanks (OR = 1.20, 95% CI: 0.16–8.98) had an odds ratio of positivity similar to geysers, highlighting similar risk across these hot water sources. Cold tap water and boreholes had substantially lower odds ratios due to small sample sizes and were not statistically significant.

The results of bivariate analysis of physico-chemical parameters indicated in Table 6 indicated that there was no significant positivity between *L. pneumophila* counts (MPN) and water temperature, chlorine levels, or turbidity (all *p* > 0.05 by Chi-square and Fisher’s exact tests). However, there was a significant correlation between positive *Legionella* and pH levels outside of the range 6.5 to 8.5, with higher odds of positivity (OR = 11.8, 95% CI: 2.0–69.9) confirmed by both the Chi-square test (*p* = 0.001) and Fisher’s exact test (*p* = 0.001), as shown in Table 6. Corrected OR 95% CI were calculated using the Haldane–Anscombe correction for sparse data.

## 4. Discussion

The presence of *L. pneumophila* was detected in 93% (*n* = 14) of the buildings sampled. The colonisation of this pathogen is well-documented in premise plumbing systems, especially in older buildings with outdated infrastructure. These buildings often experience corrosion of plumbing infrastructure materials, which releases organic nutrients that facilitate biofilm development. These conditions, combined with low disinfectant residuals, favourable temperature and pH ranges, and the overwhelming presence of free-living amoebae (FLA), support *Legionella* proliferation.

In South Africa, *Legionella* has been reported in numerous environments including hospital water systems [33], harvested rainwater systems [24,40], dental units [41], acid mine drainage [42], wastewater and treatment plants [43,44], river sediments [45], electric water heaters [27], and energy-efficient hot water systems [46], involving both urban and rural water networks [47]. This study adds high-rise single-residence buildings in Hillbrow to this growing body of knowledge. According to the National Institute for Communicable Diseases, 105 cases of Legionnaires’ disease (LD) were reported across all nine provinces between 2023 and 2025, with 42 cases in 2023, 63 in 2024, and 31 in 2025 so far. The Western Cape reported the highest incidence (22 in 2023; 19 in 2024), followed by Gauteng (15 in 2023; 19 in 2024; 12 in 2025) [48]. These figures underline the critical need for systematic *Legionella* monitoring and public health education on how to mitigate the risk of infection.

Several factors contribute to the elevated risk of *Legionella* in Hillbrow’s high-rise buildings. The design of high-rise plumbing systems with their extended pipe lengths, high surface-area-to-volume ratios, and stagnant zones promotes biofilm formation [49,50]. The risk of *Legionella* infection is higher in high-rise buildings than in one-level homes [49]. The multiple-floor infrastructure of these high-rise buildings provides a high surface-to-volume ratio, dead legs, as well as water stagnation points that promote biofilm formation and microbial growth [50,51]. Moreover, the overcrowded nature of the buildings, where individual rooms are rented out separately, strains infrastructure and may affect tenants’ hygiene practices [25]. This places pressure on resources, infrastructure and impacts the personal hygiene of individuals.

All 15 buildings (100%) employed a storage tank in their drinking water distribution. These storage tanks are commonly used as a means to curb increasing water shortages [52]. Storage tanks are known to cause a loss of disinfection and subsequently increase the pH of the water. This, coupled with the increase in iron and manganese, promotes corrosion of the material and promotes biofilm formation, thereby posing a risk of bacterial growth [53]. The position of the storage tank on the roof has an impact on water quality in that when the storage tank is not under any shade and in direct sunlight, this leads to unregulated water temperature increase that promotes microbial growth [53]. The prevailing material of the storage tank used by these buildings was plastic (93%). This creates an issue when the plastic material is leached into the water and provides organic carbon that induces microbial growth, thus raising concerns for human health [54]. The age of the building, the frequency of plumbing maintenance and the availability of water quality monitoring systems in high-rise buildings play a role in determining the risk of exposure, behaviour and the remedial action required as determined by risk assessment analysis. Convenient sampling was used in this study due to the inaccessibility and safety concerns raised by hijacked buildings; this sampling bias limits the generalizability to the entire population of Hillbrow.

The assessment of total and faecal coliforms is used as a hygienic water quality indicator. Their presence in drinking water is a sign that the water treatment measures in place are inadequate, and the distribution system is compromised (South African Drinking Water Quality Guidelines Vol 2). Only one building was positive for *E. coli*, and this may be due to water disinfectants losing their effectiveness, or interference in the water distribution that allowed for faecal contamination to enter the water supply [55]. This poses a health risk, as is highlighted by the SANS 241 guideline for drinking water, which states that drinking water should be free of faecal coliforms.

Water temperature plays a vital role in *Legionella* control. The consensus is that buildings are recommended to keep their hot water temperature above 50 °C and cold water below 20 °C because *Legionella* bacteria are known to grow in waters within these temperatures with a thermal preference of 25–45 °C [56]. Study results indicated that the temperature of water samples averaged 30 °C (min: 17.1 °C; max: 54.5 °C). Although 50 °C is the commonly recommended limit, studies have demonstrated significantly better control at >55 °C [57,58]. However, maintaining this range poses challenges for low-income tenants. While 82% of residents have access to hot water, only 52% use heaters regularly due to electricity costs. Of those, just 28% can regulate temperature, while 24% rely on central heating managed by building operators. The remaining 18% have no hot water systems. Statistical analysis from the study showed no significant correlation between *Legionella* positivity and water temperature, and this may be due to *Legionella* being able to survive in a broad range of temperatures and the limited variability presented by the small sample size.

The average pH was 7.7 (min: 5.47; max: 9.0), which fell within the SANS 241 guidelines for acceptable drinking water levels. However, a significant correlation was observed in the water samples with pH that fell outside of 6.5 and 8.5 and *Legionella* positivity. Although the acceptable range is effective in limiting microbial growth, it was still suitable for *Legionella* survival [21]. This indicates the necessity for *Legionella* monitoring to be considered in guidelines. Total chlorine levels averaged 1.07 mg/L (Min: 0 mg/L; Max: 3.22 mg/L), while free chlorine averaged 0.55 mg/L (min: 0 mg/L; max: 3.17 mg/L). This may be caused by the interruption of the water supply by adding a storage water tank that causes stagnations in the water supply, as well as the presence of a supplementary water source (borehole) that is employed by the buildings to curb water scarcity. The diminishing disinfectant levels put the tenants at risk because free chlorine levels are recommended to be at 1.0 mg/L [59] for effective *Legionella* control in a water system. Total chlorine and free chlorine levels for cold water were higher than those to hot water, and these results reiterate the need for well-informed water treatment and monitoring because the occurrence of *Legionella* and subsequent risk to LD was found to be strongly influenced by low levels of disinfectants in a water system [59]. Turbidity levels above 1 NTU are known to shield bacteria from disinfectants [60] and favour the growth of *Legionella* [61].

In this study, Legiolert detected *L. pneumophila* in 93% (14/15) of buildings, while the SANS 11731 culture method detected only 1%. The Legiolert’s use of enzyme–substrate liquid medium, specific to *L. pneumophila*, improves sensitivity and reduces interference by competing microbes [18,60]. Although the other samples were negative by Legiolert, they could still be positive for other *Legionella* species that could cause infection and still pose a health risk [61,62]. This assay also detects viable but non-culturable (VBNC) cells, unlike the SANS method, which relies on colony morphology and technician skill [16].

The culture method has been known to have low sensitivity when working with environmental samples, as compared to Legiolert, which has a 99% higher sensitivity for *L. pneumophila* [63]. The Legiolert method used a Quanti Tray system, who’s design of individual wells limits interaction and reduces growth inhibition [64].

The conventional PCR and real-time PCR both targeted the presence of *L. pneumophila* using the *mip* gene, which is used for the identification of *L. pneumophila* and a strain that displays biofilm-forming ability, which is crucial for the bacterium’s virulence [65]. Both PCR assays detected the gene in 13 of the 31 samples tested. Legiolert trays were sequenced and confirmed the presence of *L. pneumophila* [49]. The comparative difference in sensitivity between the Legiolert, q-PCR and the standard culture methods was similarly observed in potable water samples, with relative detection figures of 22% for Legiolert, 17% for q-PCR and 6% for the culture method [61].

Similar colonisation rates were reported with 67% in South African domestic water [49]. Higher counts of 88.2% were found in buildings in Jordan [6], 92.1% in residential buildings of Latvia [66], and 100% in other domestic buildings [67], as well as 73% in cold water taps in the USA [68].

Of the 17–29 °C cold-water samples, 65% (20/31) tested positive, compared to 35% (11/31) of the 30–55 °C warm-water samples. This aligns with studies detecting *L. pneumophila* in water as cool as 18 °C [49,59]. Similarly, a study detected more *Legionella* in their cold water (52%) than in their hot water (44%) samples [66]. The growth of this organism in a cold-water source (17 °C) is due to the ability of the bacterium to survive cold temperatures for long periods of time and only proliferate when temperatures are optimum [69]. The bacterium’s persistence in cold water underscores its adaptability and long-term survival capacity [70].

The link between FLA and *L. pneumophila* is well-established. The presence/absence of the bacteria in a system is significantly related to the presence/absence of FLA in a water system [71,72]. Thus, FLA enhances *Legionella* survival, virulence and resistance to disinfectants by providing a protective barrier for the bacteria [23,33,72]. In the present study, 10% (4/42) of isolates were identified as amoeba-associated *Legionella* by PCR, and 3 of these isolates were confirmed as *L. pneumophila* by sequencing. This is consistent with previous findings of amoeba-associated *Legionella* of 9.9% in hospital water networks [33] and 35% in residential waters in Johannesburg [49].

The sequence alignment of the samples to registered strains indicates conclusive proof of the presence of pathogenic bacterial strains in water found in high-rise buildings of Hillbrow, Johannesburg. Sequencing results showed a 96–99% similarity with *L. pneumophila*, the most clinically relevant species known to cause Legionnaires’ disease. One isolate displayed *Legionella*-like morphology on the agar plate but was not positive by PCR. This could be a non-*pneumophila* strain, such as *L. anisa,* which is also known to be found in aquatic environments. Although less commonly associated with disease than *L. pneumophila*, *L. anisa* has been documented in clinical cases and environmental sources, and its presence suggests ecological diversity of *Legionella* in the sampled environments [73]. This result indicates a potentially uncategorized or less-studied strain, and while it does not confirm species-level identification, it still represents a member of the genus *Legionella* with probable environmental persistence. These results, therefore, validate the use of the *mip* gene as a molecular marker for species-level identification. Moreover, the co-detection of amoeba-associated strains and VBNC cells using Legiolert and molecular tools further indicates the complexity and potential virulence of the microbial communities in these systems.

## 5. Conclusions

The study’s findings indicate a high occurrence of *L. pneumophila* in the buildings examined, underscoring a prevalent public health concern. The presence of more *L. pneumophila* in low-temperature water samples as compared to the higher-temperature samples defies common knowledge of the pathogen and indicates a need for revised water control measures. Due to socio-economic factors, most apartments/dwellings decided not to use their geysers to heat water, but this does not seem to exempt them from the risk of the bacterium. To better prevent and control the occurrence of disease, water samples need to be constantly monitored.

The overwhelming presence of free-living *Amoebae* found in water and biofilm samples and the discovery of amoeba-associated *Legionella* exacerbates the health risk of *Legionella* infection amongst other premise plumbing pathogens and renders normal antibacterial countermeasures ineffective. The gap between the number of reported cases of LD and the sporadic sources of *Legionella* investigation in South Africa needs to be resolved to prevent further cases originating from a single source that may lead to LD outbreaks.

## Figures and Tables

**Table 1:** Summary of collected water and swab samples.

Sample Type		Number of Samples Collected
Water (*n* = 67)	Hot water (30–55 °C)	23 (34%)
	Cold water (17–29 °C)	44 (66%)
Swabs (*n* = 121)	Bathroom faucet	67 (56%)
	Showerhead	11 (9%)
	Toilet Bowl	39 (32%)
	Storage tank	4 (3%)
Total number of samples (*n* = 188)		

**Table 3:** *Legionella* results described by sample type (water and swabs) from each method used. Note: n/a means the method was not applicable because the method was validated for water samples only, and PCR was performed only on positive samples.

Detection Method		Water Samples (*n* = 67)				Swab Samples (*n* = 121)	
		Hot Water (*n* = 23)		Cold Water (*n* = 44)			
		*Legionella* Positive	*Legionella* Negative	*Legionella* Positive	*Legionella* Negative	*Legionella* Positive	*Legionella* Negative
Culture (SANS 11731:2017)	BCYE agar	0% (0/23)	100% (23/23)	0% (0/44)	100% (44/44)	0% (0/67)	100% (67/67)
	Legiolert	48% (11/23)	52% (12/23)	45% (20/44)	55% (24/44)	n/a	n/a
PCR	*Legionella* spp.	18% (2/11)	82% (9/11)	55% (11/20)	45% (9/20)	n/a	n/a
	*L. pneumophila*	18% (2/11)	82% (9/11)	55% (11/20)	58% (18/31)	n/a	n/a
Real-time PCR	*L. pneumophila*	18% (2/11)	82% (9/11)	55% (11/20)	58% (18/31)	n/a	n/a
Amoeba-associated *Legionella*	BCYE agar	9% (2/23)	91% (21/23)	0% (0/44)	100% (67/67)	2% (2/121)	98% (119/121)

**Table 4:** Blast analysis of sequence alignment for *L. pneumophila*.

Isolate	Description	Query Cover	E-Value	% Identity	Accession Number
1	*Legionella pneumophila* strain NCTC12180 genome assembly, chromosome: 1	96%	1 × 10^−50^	100	LR133933.1
2	*Legionella pneumophila* strain SPF585 chromosome, complete genome	93%	4 × 10^−46^	99.09	CP174240.1
3	*Legionella pneumophila* strain C11_O chromosome, complete genome	99%	2 × 10^−43^	94.83	CP015945.1

**Table 5:** Positive *Legionella* in water samples per source (geyser, boiler, storage tank, cold water taps and borehole) (Wilson 95% Confidence Interval, odds ratios vs. geyser as reference).

Sample Type	Detection Method	Samples Tested	Samples Positive	% Positive	95% CI	Odds Ratio vs. Geyser (95% CI)
Geyser	Legiolert (MPN/100 mL)	45	21	46.7	32.9–60.9	Reference
Boiler	Legiolert (MPN/100 mL)	12	6	50.0	25.4–74.6	1.14 (0.34–3.80)
Storage Tank	Legiolert (MPN/100 mL)	4	2	50.0	15.0–85.0	1.20 (0.16–8.98)
Cold tap	Legiolert (MPN/100 mL)	5	1	20.0	3.6–62.4	0.31 (0.03–2.94)
Borehole	Legiolert (MPN/100 mL)	1	0	0.0	0.0–79.3	0.17 (0.01–4.54)

**Table 6:** Association of environmental parameters with *L. pneumophila* positivity (bivariate analysis). Odds ratios (ORs) with 95% CI were performed using the Haldane–Anscombe correction (adding 0.5) when zero cell counts were present. Chi-square tests were applied when frequencies ≥ 5; otherwise, Fisher’s exact test was used when expected counts < 5.

Parameter	N Negative	N Positive	OR (95% CI)	Chi-Square (χ^2^) *p*-Value	Fisher’s Exact *p*-Value
Temperature < 50 °C	34 vs. 32	1 vs. 1	1.06 (0.11–10.73)	0.966	1.000
Free chlorine < 0.5 mg/L	19 vs. 16	16 vs. 17	1.25 (0.49–3.21)	0.632	0.808
pH outside 6.5–8.5	34 vs. 22	1 vs. 11	11.8 (2.0–69.9)	0.001	0.001
Turbidity > 1 NTU *	35 vs. 32	0 vs. 1	3.3 (0.13–83.3)	0.299	0.485

* Odds ratios (ORs) with 95% CI was done using the Haldane–Anscombe correction (adding 0.5) when zero cell counts were present. Chi-square test was applied when frequencies were ≥5; otherwise, Fisher’s exact test was used when expected counts were <5.

## Data Availability

The original contributions presented in this study are included in the article/Supplementary Materials. Further inquiries can be directed to the corresponding author.

## Supplementary Materials

The following supporting information can be downloaded at https://www.mdpi.com/article/10.3390/microorganisms13092152/s1, Table S1: Building (*n* = 15) characteristics for each building (H1 to H15), including the number of apartment and floors, how occupied the building is (in %), the availability of hot water heating equipment, storage tank (use, location and material); Table S2: physiochemical parameters of the water samples including Temperature, pH, free and total chlorine, turbidity, and total dissolved solutes.
