# Supplementary material for: Legionella in the City: Unveiling Legionella pneumophila in Hillbrow’s High-Rise Water Systems
Source: Microorganisms. 2025 Sep 15;13(9):2152. doi: 10.3390/microorganisms13092152 (PMC12472198; doi:10.3390/microorganisms13092152)
Supplement: Supplementary file 1 [file microorganisms-13-02152-s001.zip › microorganisms-3814200-supplementary.pdf]

Table S1. Building (n = 15) characteristics for each building (H1 to H15), including the number of apartment and floors, how occupied the building is (in %), the availability of hot water heating equipment, storage tank (use, location and material);

Building	Apartments per building	Floors per building	Building occupancy	Water heating equipment	Secondary water treatment	Water quality monitoring	Storage Tank		
							Material	Usage	Location
HB1	6	10	50%	N/A	No	Monthly	Plastic	Drinking/general use	Ground floor
HB2	5	8	97%	N/A	No	Monthly	Plastic	Drinking/general use	Rooftop
HB3	49	5	50%	Geyser	When necessary	Not done	Plastic	Drinking/general use	Rooftop
HB4	113	14	50%	Boiler	Onsite	Monthly	Plastic	Drinking/general use	Ground floor
HB5	6	8	65%	Geyser	Onsite	Not done	Plastic	Drinking/general use	Rooftop
HB6	15	10	90%	Boiler	Onsite	Monthly	Plastic	Drinking/general use	Rooftop
HB7	26	8	65%	Geyser	No	Not done	Plastic	Drinking/general use	Rooftop
HB8	170	9	100%	Geyser	No	Not done	Plastic	Drinking/general use	Ground floor
HB9	27	4	50%	Geyser	Onsite	Monthly	Plastic	Drinking/general use	Rooftop
HB10	40	6	65%	Geyser	No	Not done	Plastic	Drinking/general use	Ground floor
HB11	51	5	90%	Geyser	No	Not done	Plastic	Drinking/general use	Ground floor
HB12	147	8	65%	Geyser	No	Not done	Plastic	Drinking/general use	Rooftop
HB13	27	6	65%	Geyser	No	Not done	Plastic	Drinking/general use	Rooftop
HB14	71	5	50%	Geyser	No	Monthly	Plastic	Drinking/general use	Ground floor
HB15	8	14	95%	Geyser	Onsite	Not done	Metal	Drinking/general use	Rooftop

Table S2 Chlorine and temperature readings for the buildings sampled

	Building Name	Free chlorine	Total Chlorine (mg/L)	Temperature °C
**Hot Water**	HB4	1,02	1,18	28,85
	HB6	0,99	1,35	30,7
	HB7	0,27	0,48	47,88
	HB8	0,13	0,91	40,2
	HB10	0,02	0,02	44,54
	HB11	0	0,64	44,17
	HB12	0,13	0,33	38,74
	HB14	0,3	1,18	30,15
**Cold water**	HB1	0	0	21,25
	HB2	2,1	2,83	21,9
	HB3	1,02	1,92	18,2
	HB5	0,65	2,38	18,77
	HB9	0	0	24,85
	HB13	0,9	1,3	21,4
	HB15	0,66	1,58	19,37
